# Divergent evolutionary dynamics of benign and malignant tumors

**DOI:** 10.1073/pnas.2519203122

**Published:** 2025-11-06

**Authors:** George Butler, Joanna Baker, Sarah R. Amend, Kenneth J. Pienta, Chris Venditti

**Affiliations:** ^a^University College London Cancer Institute, University College London, London WC1E 6DD, United Kingdom; ^b^Cancer Ecology Center, The Brady Urological Institute, Johns Hopkins School of Medicine, Baltimore, MD 21287; ^c^School of Biological Science, University of Reading, Reading RG6 6AS, United Kingdom; ^d^School of Life, Health, and Chemical Sciences, The Open University, Walton Hall, Milton Keynes MK7 6AA, United Kingdom

**Keywords:** cancer evolution, comparative phylogenetics, comparative oncology

## Abstract

Benign and malignant (cancerous) tumors differ markedly in their impact on organismal fitness, yet studies in comparative oncology rarely distinguish between them. Using a Bayesian phylogenetic framework across birds and mammals, we show that while both tumor types increase in prevalence with body mass, only the prevalence of malignant tumors is negatively associated with the rate of body size evolution—suggesting that adaptive mechanisms of cancer defense are associated with rapidly evolving lineages. Additionally, the rate of lineage diversification is positively associated with the prevalence of both tumor types in birds but not mammals, potentially reflecting differences in genome architecture and speciation dynamics. Together, these results highlight distinct macroevolutionary drivers of benign versus malignant tumor prevalence and underscore the value of treating tumor types separately in comparative oncology.

Tumors (or neoplastic growths) are typically classified as either benign (noncancerous) or malignant (cancerous) (1). While both are instances of abnormal cellular proliferation, malignant tumors pose a greater threat to survival owing to their ability for tissue invasion and metastasis (spread of cancer to distal sites within the body). From an evolutionary perspective, this distinction is critical—benign tumors are expected to have limited impact on fitness, while malignancies could significantly reduce reproductive success (2).

Recent comparative analyses testing the evolutionary basis of cancer prevalence across species have tended to focus on associations with life history—using traits such as body size (3), gestation length (4), reproductive output (5), and lifespan (6). Moreover, comparative evolutionary studies exploring neoplasia prevalence have not distinguished between benign and malignant tumors. Given the stark difference in potential survival, we hypothesize that benign and malignant tumor prevalence would exhibit divergent associations with key evolutionary processes.

Two such processes—the rate of species diversification and the rate of body size evolution—reflect macroevolutionary dynamics (7) that may influence tumor prevalence. Previous work has shown that faster rates of body size evolution are associated with lower malignancy prevalence in birds and mammals, even after accounting for size (3). This has been interpreted as evidence of adaptive cancer resistance mechanisms emerging alongside evolutionary increases in body size. However, whether benign tumor prevalence shows a similar pattern remains unknown. Likewise, while high diversification rates may signify “evolutionary success,” their relationship to tumor burden has never been tested.

## Results

Here, we use a Bayesian multivariate phylogenetic generalized linear mixed model (MPGLMM) (8) to investigate how the prevalence of benign and malignant tumors varies in relation to body mass, the rate of body mass evolution (henceforth referred to as path-wise rate), and the rate of lineage diversification across birds and mammals (*Materials and Methods*). We model tumor prevalence as a function of these variables, controlling for the number of necropsies per species, and testing for shared and lineage-species effects (*Materials and Methods*).

**Fig. 1.**
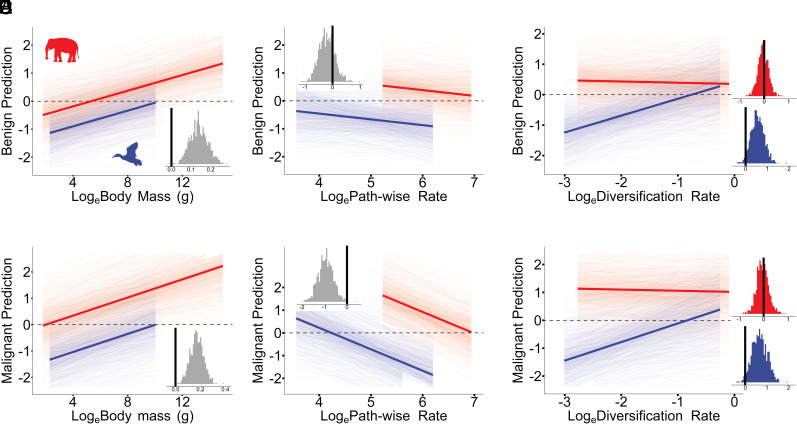
The evolutionary dynamics of benign and malignant growths across birds (blue) and mammals (red). In all cases, the posterior predicted slopes are plotted, and the mean average predicted slopes are imposed. Insets show the posterior distribution of the estimated slopes, where black vertical lines indicate 0 on the x-axis. A slope is significant if less than 5% of the posterior distribution crosses 0 (*P*_x_ < 0.05). The dotted horizontal line indicates y = 0. Body mass is positively associated with the prevalence of (*A*) benign (*P*_x_ = 0.001) and (*B*) malignant (*P*_x_ = 0.002) tumors. In contrast, the path-wise rate (*Materials and Methods*) is not associated with (*C*) benign tumor prevalence (*P*_x_ = 0.230) but is negatively associated with (*D*) malignant tumor prevalence (*P*_x_ = 0.002). Furthermore, (*E*) benign tumor prevalence is positively associated with diversification rate in birds (*P*_x_ = 0.027) but not in mammals (*P*_x_ = 0.415). Likewise, (*F*) malignant tumor prevalence is positively associated with diversification rate in birds (*P*_x_ = 0.022) but not in mammals (*P*_x_ = 0.426).

We found that both benign and malignant tumor prevalence increase with body mass across birds and mammals (*P*_x_ = 0.001 and 0.002, β = 0.140 and 0.172, CI = 0.055 to 0.231 and 0.059 to 0.270, Fig. 1 *A* and *D*). However, only malignant tumor prevalence was negatively associated with path-wise rate (*P*_x_ = 0.230 and 0.002, β = −0.210 and −0.944, CI = −0.760 to 0.356 and −1.590 to −0.301, Fig. 1 *B* and *E*). This pattern supports the hypothesis that natural selection has favored adaptations to suppress malignant transformation in species undergoing rapid body size evolution, while benign tumors—owing to the fact that they are likely to be less detrimental—may not experience similar selective pressures. The lack of association with benign tumors suggests that the protective mechanisms involved may be specific to processes such as metastasis, rather than dysregulation of cellular proliferation (2).

On the other hand, the rate of lineage diversification in birds, was positively associated with the prevalence of both benign and malignant tumors (*P*_x_ = 0.027 and 0.022, β = 0.554 to 0.667, CI = 8.80e-05 to 1.155 and 0.025 to 1.316, Fig. 1*C*). No such relationship was found in mammals (*P*_x_ = 0.415 and 0.426, β = −0.040 and −0.041, CI = −0.458 to 0.372 and −0.498 to 0.472, Fig. 1*F*). This discrepancy may reflect fundamental differences in genome architecture. Generally, birds possess smaller, more compact genomes than mammals (9), potentially making them more susceptible to tumor-promoting genomic instability associated with speciation processes, such as chromosomal rearrangements (10).

**Fig. 2.**
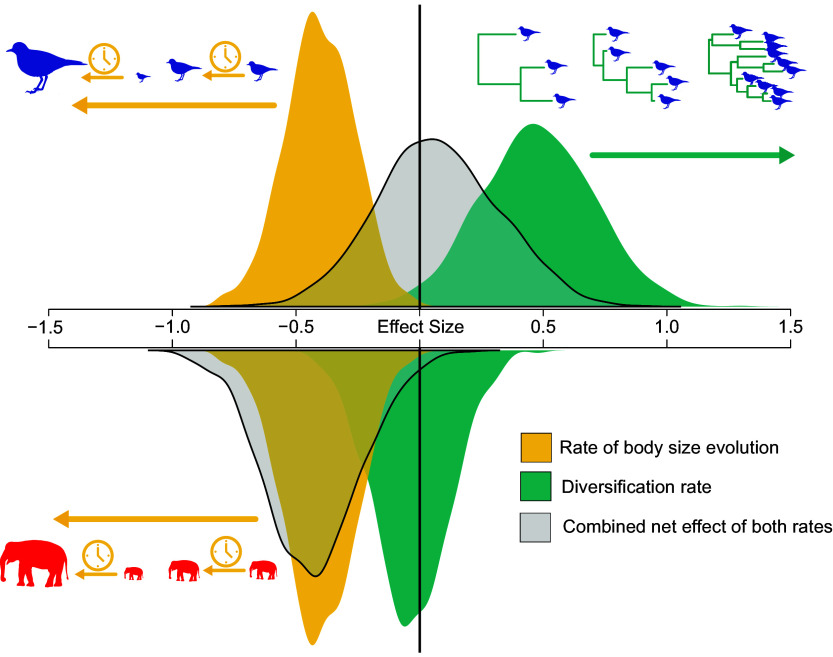
Posterior distributions for the estimated standardized effect size for the rate of body size evolution and diversification rate on malignancy prevalence in birds (blue) and mammals (red). The same significant negative effect for the rate of body size evolution on malignancy prevalence is estimated for birds and mammals (yellow). A significant positive effect of diversification rate on malignancy prevalence is estimated for birds, but a nonsignificant effect is estimated in mammals (green). In birds, the combined net effect of both covariates is approximately 0, whereas in mammals, there is a combined net negative effect on malignancy prevalence (gray overlays).

These contrasting effects—a negative association with body size evolution and a positive association with diversification—suggest that tumor prevalence in birds may be influenced by a tension between adaptive change to reduce malignancy and stochastic genomic changes increasing tumor risk, resulting in a net neutral effect on malignancy prevalence. In contrast, the nonsignificant effect of lineage diversification in mammals illustrates the lineage-specific nature of evolutionary dynamics that shape tumor prevalence and the net negative effect on malignancy prevalence (Fig. 2). Nevertheless, tumor prevalence remains beholden to the effect of body size in both birds and mammals.

Overall, our findings emphasize that benign and malignant tumors are not only clinically distinct but also evolutionarily divergent. Malignancies, owing to their propensity to directly influence survival, appear to be constrained by stronger selective forces, while benign tumors persist relatively unconstrained. Studying both tumor types in tandem provides a more nuanced view of how evolutionary processes shape cancer susceptibility, offering new insights into the origins of cancer resistance mechanisms across the tree of life, and more importantly, in combating the emergence of therapy resistance and treatment failure in humans.

## Materials and Methods

Tumor prevalence data for each species and the phylogenetic tree are available from Compton et al. (4). Benign tumor prevalence was quantified as the difference in neoplasia and malignant tumor prevalence for each species. Body size data, posterior distributions for body size rate-scaled phylogenetic trees, and lineage diversification rate-scaled trees are available from Cooney and Thomas (7). The amount of body size evolution (path-wise rate) was quantified as outlined in Butler et al. (3). The rate of lineage diversification (speciation rate – extinction rate) was quantified as the average weighted distance from root to tip of the diversification rate-scaled trees as outlined in Cooney and Thomas. MPGLMMs were fitted as outlined in Butler et al. (3). Briefly, MPGLMMs were fitted in a Bayesian Markov Chain Monte Carlo (MCMC) framework using the MCMCglmm R package (8). Using a process of forward stepwise selection, separate slopes were estimated for each class and each dependent variable. If no significant difference was found, then a separate slope was fitted across birds and mammals. In the final model, a single slope was estimated across birds and mammals for the number of necropsies, body size, and path-wise rate, and separate slopes were estimated for the rate of lineage diversification for each class. Regression parameter significance was assessed by the proportion of the posterior distribution that crosses zero (*P*_x_), where *P*_x_ < 0.05 is considered to be significantly different from 0. The necessary code to fit the MPGLMMs and the fitted model is available at https://github.com/george-butler/divergent_evo_dynamics.

## Data Availability

Code to fit the MPGLMMs and the fitted model data have been deposited in GitHub (https://github.com/george-butler/divergent_evo_dynamics). Previously published data were used for this work (4, 7).
